# Supplementary material for: Enhanced production of heterologous proteins by a synthetic microbial community: Conditions and trade-offs
Source: PLoS Comput Biol. 2020 Apr 13;16(4):e1007795. doi: 10.1371/journal.pcbi.1007795 (PMC7179936; doi:10.1371/journal.pcbi.1007795)
Supplement: S1 Fig — (PDF) [file pcbi.1007795.s001.pdf]

S1 Fig – Results of the *a-posteriori* parameter identifiability analysis*

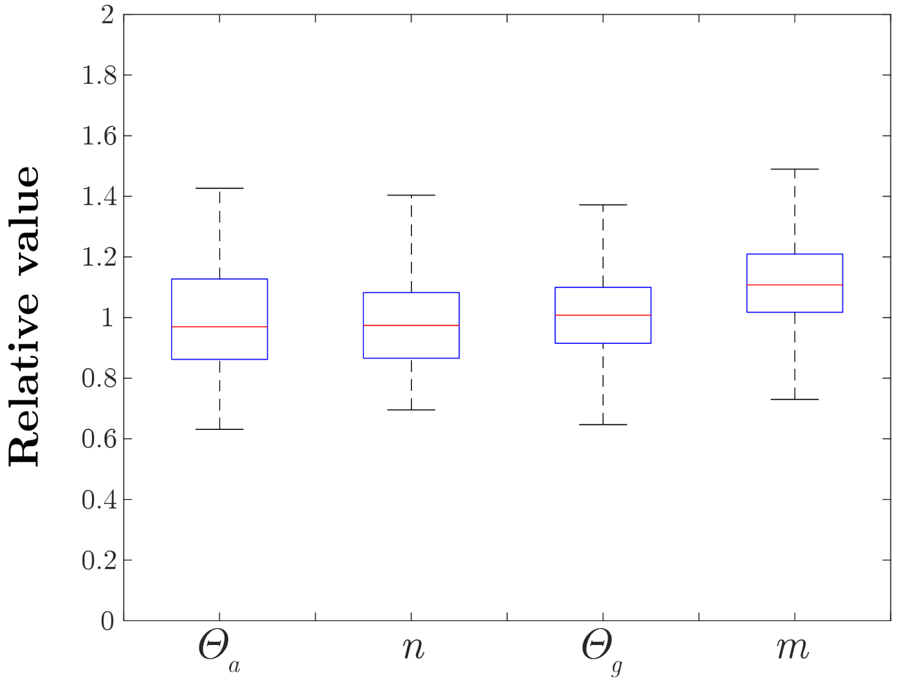

Result of the *a-posteriori* identifiability analysis using a procedure analogous to bootstrapping as described in [1]. We generated 1000 new datasets from the experimental dataset of Fig. 2C [2] by adding to each one of the estimated data points a quantity randomly sampled from the set of residuals obtained by comparing the model predictions and the experimental data. The resulting bootstrap datasets were used to fit the model and obtain new values for Θ_a, n, Θ_g and m. The box plot shows the statistic resulting from this procedure. Data are normalized with respect to the estimated value reported in Table 1. The central line of each box represents the median, the bottom and the top edges correspond to the lower and upper quartiles, respectively, and the whiskers extend from 1.5 IQR (interquartile range) below the lower quartile to 1.5 IQR above the upper quartile (see the *boxplot* function in Matlab).

Supporting references

[1] Stefan D, Pinel C, Pinhal S, Cinquemani E, Geiselmann J, de Jong H. Inference of quantitative models of bacterial promoters from time-series reporter gene data. PLoS Comput Biol. 2015;11(1):e1004028.

[2] Enjalbert B, Millard P, Dinclaux M, Portais JC, Létisse F. Acetate fluxes in *Escherichia coli* are determined by the thermodynamic control of the Pta-AckA pathway. Sci Rep. 2017;7:42135.

*Supporting Information of "Enhanced production of heterologous proteins by a synthetic microbial community: Conditions and trade-offs" (M. Mauri, J.-L. Gouzé, H. de Jong, E. Cinquemani)
