# Supplementary material for: Enhanced production of heterologous proteins by a synthetic microbial community: Conditions and trade-offs
Source: PLoS Comput Biol. 2020 Apr 13;16(4):e1007795. doi: 10.1371/journal.pcbi.1007795 (PMC7179936; doi:10.1371/journal.pcbi.1007795)
Supplement: S2 Fig — (PDF) [file pcbi.1007795.s002.pdf]

S2 Fig – Model predictions for the producer strain*

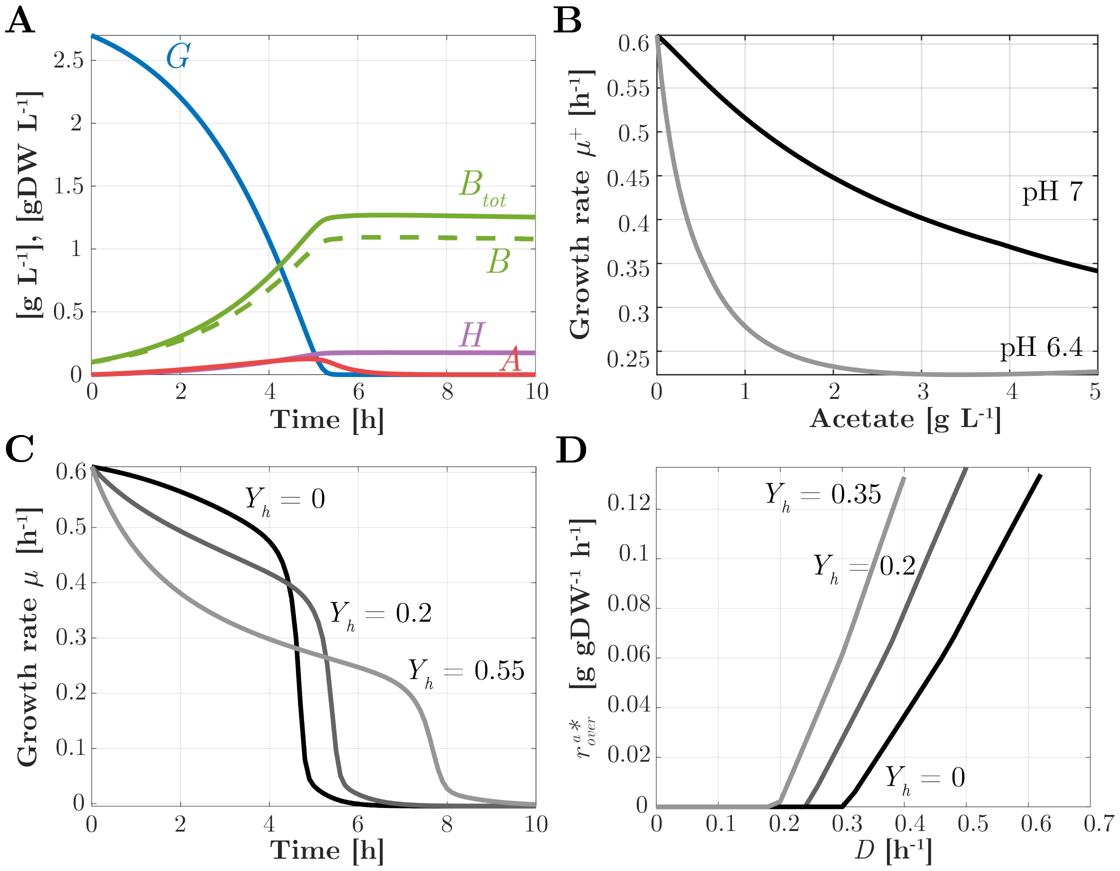

Model predictions for the producer strain. (A) Time evolution of the concentration of glucose G (blue curve), acetate A (red curve), heterologous protein concentration H (violet curve), autocatalytic biomass B (green dashed curve) and total biomass $B_{tot} = B + H$ (green solid curve) in a bioreactor operating in batch mode, with initial concentrations of 2.7 g L^{-1} of glucose and 0.1 g L^{-1} of biomass. (B) Effect of increasing concentrations of acetate in the medium on the exponential growth rate of the bacteria, in the same conditions as in panel A, with the acetate inhibition coefficient Θ_a set equal to 3.5 g L^{-1} and 0.5 g L^{-1}, corresponding to pH 7 and 6.4, respectively. (C) Effect of metabolic load on the time-varying growth rate in the same conditions as in panel A, obtained by overexpressing the heterologous protein H for three different yield coefficients $Y_h = 0$, $Y_h = 0.15$, and $Y_h = 0.55$. (D) Effect of overexpression of heterologous protein H on the onset of acetate overflow, indicated by the overflow rate r^*_{over} reached at steady state in a bioreactor operating in continuous mode, with $G_{in} = 20$ g L^{-1}, for three different yield coefficients ($Y_h = 0$, $Y_h = 0.15$, $Y_h = 0.25$), and $G_{in} = 20$ g L^{-1}.

*Supporting Information of "Enhanced production of heterologous proteins by a synthetic microbial community: Conditions and trade-offs" (M. Mauri, J.-L. Gouzé, H. de Jong, E. Cinquemani)
