# Supplementary material for: Enhanced production of heterologous proteins by a synthetic microbial community: Conditions and trade-offs
Source: PLoS Comput Biol. 2020 Apr 13;16(4):e1007795. doi: 10.1371/journal.pcbi.1007795 (PMC7179936; doi:10.1371/journal.pcbi.1007795)
Supplement: S3 Fig — (PDF) [file pcbi.1007795.s003.pdf]

S3 Fig – Example simulation of the consortium in conditions leading to coexistence*

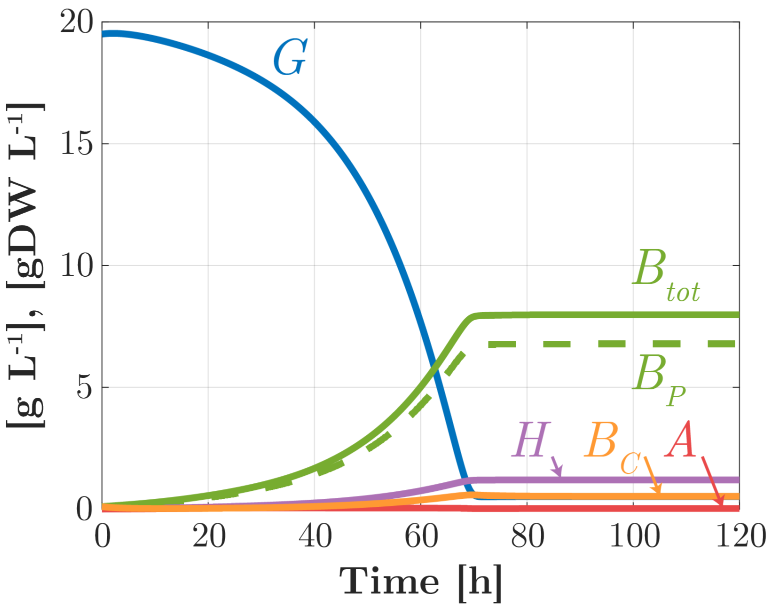

Example simulation of the consortium in conditions leading to coexistence with initial conditions $B_{P0} = 0.1$ gDW L^{-1}, $B_{C0} = 0.1$ gDW L^{-1}, $G_0 = 19.5$ g L^{-1}, and all other initial values set to 0.

*Supporting Information of "Enhanced production of heterologous proteins by a synthetic microbial community: Conditions and trade-offs" (M. Mauri, J.-L. Gouzé, H. de Jong, E. Cinquemani)
