# Supplementary material for: Enhanced production of heterologous proteins by a synthetic microbial community: Conditions and trade-offs
Source: PLoS Comput Biol. 2020 Apr 13;16(4):e1007795. doi: 10.1371/journal.pcbi.1007795 (PMC7179936; doi:10.1371/journal.pcbi.1007795)
Supplement: S4 Fig — (PDF) [file pcbi.1007795.s004.pdf]

S4 Fig – Explicit computation and analysis of the roots of the multinomial equations characterizing the steady states of the consortium*

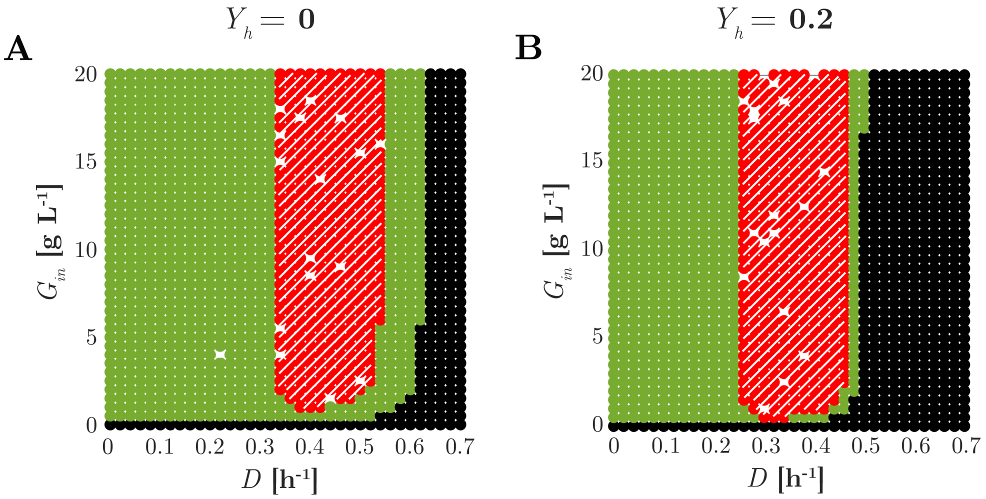

Explicit computation and analysis of the roots of the multinomial equations characterizing the steady states of the consortium obtained as described in *Methods* for $Y_h = 0$ (A) and $Y_h = 0.2$ (B). In green, hatched red and black, the region where only the producer, both producer and cleaner, and none of the two strains are present at steady state, respectively. The *psolve* routine of Matlab [1] was not always able to produce the correct solution due to numerical issues, as shown by the sparse blanks.

Supporting references

[1] Zeng Z, Li TY. NAClab: a Matlab toolbox for numerical algebraic computation. ACM Comm Comp Algebra. 2013;47(3-4):170–3.

*Supporting Information of "Enhanced production of heterologous proteins by a synthetic microbial community: Conditions and trade-offs" (M. Mauri, J.-L. Gouzé, H. de Jong, E. Cinquemani)
