# Supplementary material for: Enhanced production of heterologous proteins by a synthetic microbial community: Conditions and trade-offs
Source: PLoS Comput Biol. 2020 Apr 13;16(4):e1007795. doi: 10.1371/journal.pcbi.1007795 (PMC7179936; doi:10.1371/journal.pcbi.1007795)
Supplement: S5 Fig — (PDF) [file pcbi.1007795.s005.pdf]

S5 Fig – Analysis of the unique stable steady state of the consortium, and corresponding productivity heatmap, as a function of D and Y_h*

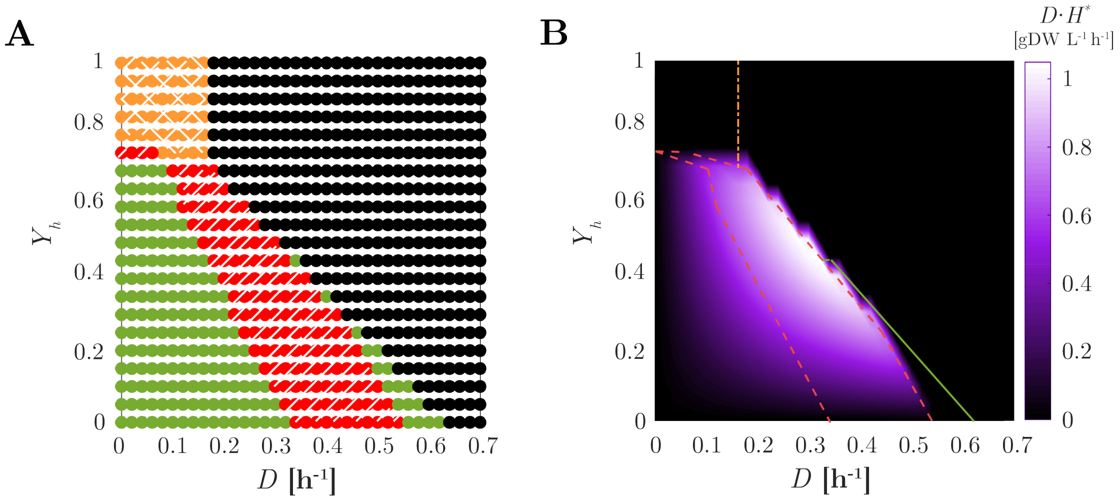

(A) Analysis of the unique stable steady state of the consortium as a function of D and Y_h. In hatched red: stable coexistence; in green: stable existence of the producer only; in doubly-hatched orange: stable existence of the cleaner only; in black: washout of both strains. (B) Heatmap of the productivity of the consortium ($D\,H^*$) as a function of D and Y_h. The boundaries of the domains of coexistence, of existence of the sole producer, and of the sole cleaner are reported from Fig. S5A in dashed red, solid green, and dahs-dotted orange lines, respectively.

*Supporting Information of "Enhanced production of heterologous proteins by a synthetic microbial community: Conditions and trade-offs" (M. Mauri, J.-L. Gouzé, H. de Jong, E. Cinquemani)
