# Supplementary material for: Enhanced production of heterologous proteins by a synthetic microbial community: Conditions and trade-offs
Source: PLoS Comput Biol. 2020 Apr 13;16(4):e1007795. doi: 10.1371/journal.pcbi.1007795 (PMC7179936; doi:10.1371/journal.pcbi.1007795)
Supplement: S6 Fig — (PDF) [file pcbi.1007795.s006.pdf]

S6 Fig – Acetate overflow rate r^{a*}_{over} and uptake rate r^{a*}_{up} as a function of the dilution rate D, for the consortium in steady state in the same conditions as in Fig. 5*

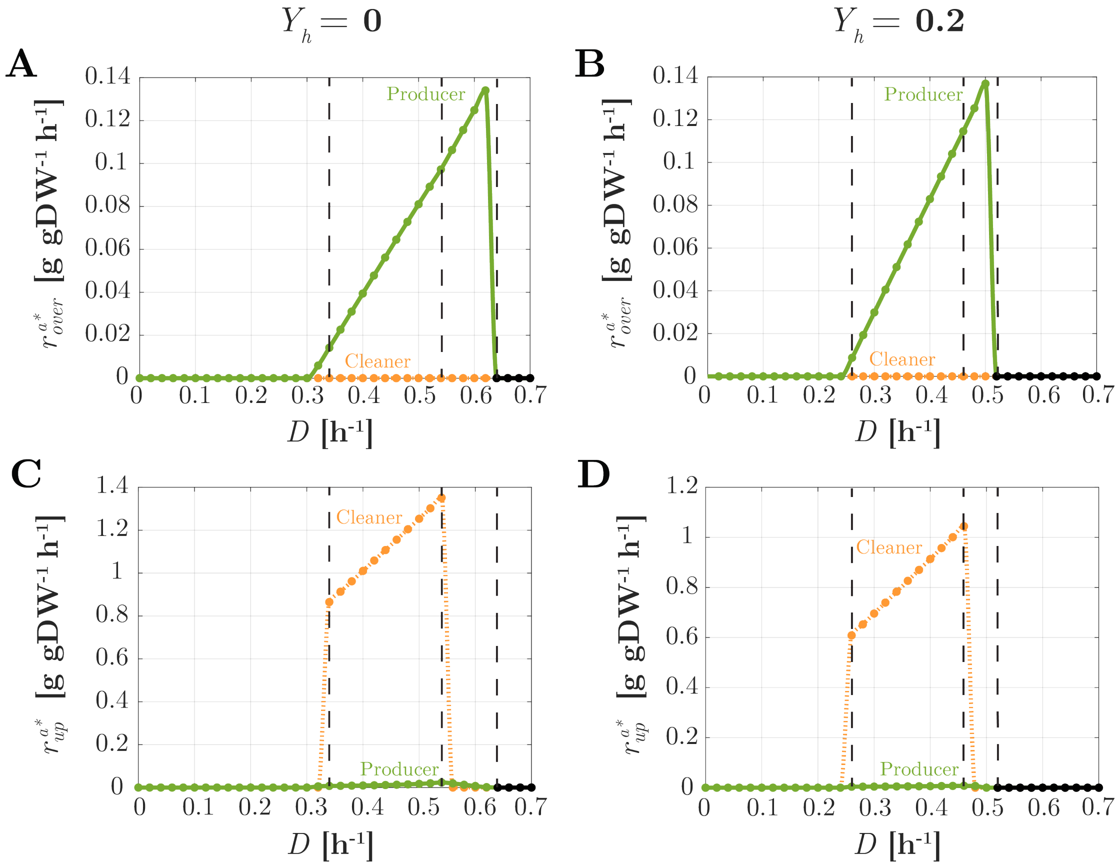

Acetate overflow rate r^{a*}_{over} (A)-(B) and acetate uptake rate r^{a*}_{up} (C)-(D) as a function of the dilution rate D, for the consortium in steady state in the same conditions as for Fig. 5 in main text ($G_{in} = 20$ g L^{-1}). Left panels: $Y_h = 0$; right panels: $Y_h = 0.2$. Green dots with solid lines: producer rates; orange dots with dotted lines: cleaner rates. Black denotes absence (washout) of both strains.

*Supporting Information of "Enhanced production of heterologous proteins by a synthetic microbial community: Conditions and trade-offs" (M. Mauri, J.-L. Gouzé, H. de Jong, E. Cinquemani)
