# Supplementary material for: Enhanced production of heterologous proteins by a synthetic microbial community: Conditions and trade-offs
Source: PLoS Comput Biol. 2020 Apr 13;16(4):e1007795. doi: 10.1371/journal.pcbi.1007795 (PMC7179936; doi:10.1371/journal.pcbi.1007795)
Supplement: S7 Fig — (PDF) [file pcbi.1007795.s007.pdf]

S7 Fig – Total steady-state biomass production in chemostat ($Y_h = 0.2$) as a function of D for the consortium and for a producer growing in isolation*

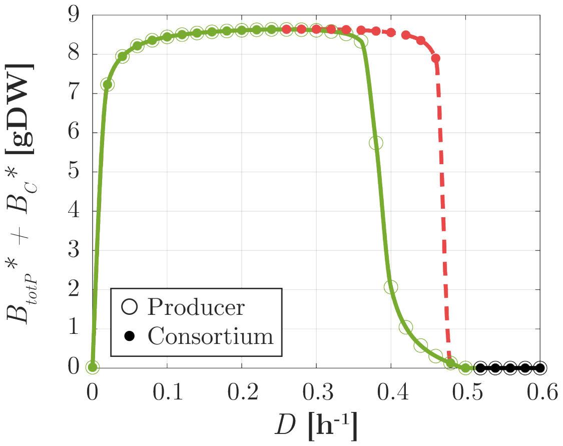

Total steady-state biomass production ($B^*_{totP} + B^*_C$) in chemostat ($Y_h = 0.20$) for $G_{in} = 20$ g L^{-1} as a function of D for the consortium (filled circles), and for a producer growing in isolation (empty circles), as in Fig. 6. In dashed red: stable coexistence; in solid green: stable existence of the producer only; in black: washout of both strains.

*Supporting Information of "Enhanced production of heterologous proteins by a synthetic microbial community: Conditions and trade-offs" (M. Mauri, J.-L. Gouzé, H. de Jong, E. Cinquemani)
