# Supplementary material for: Enhanced production of heterologous proteins by a synthetic microbial community: Conditions and trade-offs
Source: PLoS Comput Biol. 2020 Apr 13;16(4):e1007795. doi: 10.1371/journal.pcbi.1007795 (PMC7179936; doi:10.1371/journal.pcbi.1007795)
Supplement: S8 Fig — (PDF) [file pcbi.1007795.s008.pdf]

S8 Fig – Performance of the heterologous protein production process in chemostat ($Y_h = 0.2$), in the case where the cleaner uptake rate of glucose is fixed to 0^*

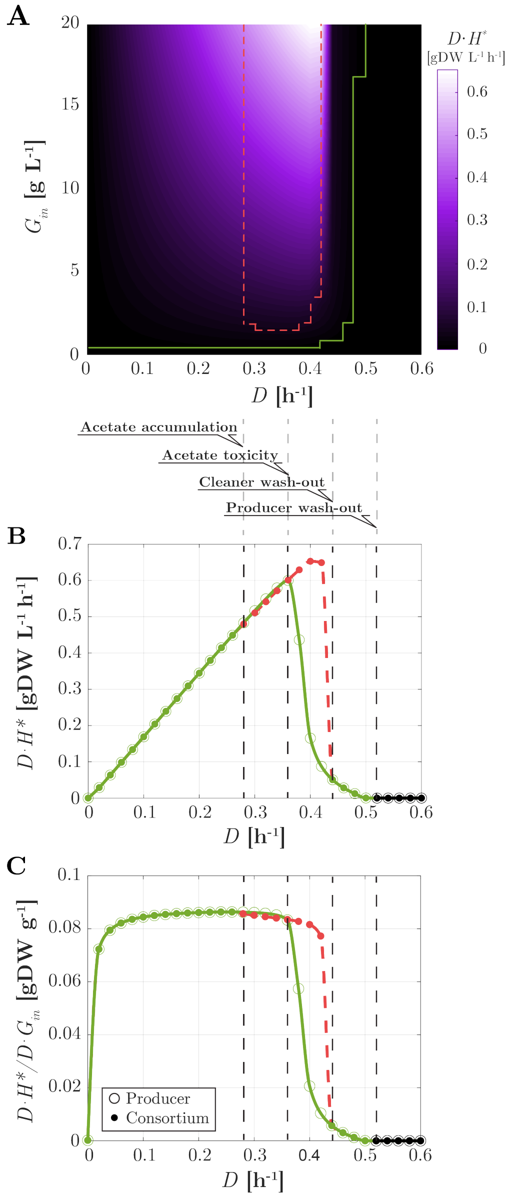

Performance of the heterologous protein production process in chemostat ($Y_h = 0.2$), in the case where the cleaner uptake rate of glucose is fixed to 0. (A) Heatmap of the productivity of the consortium (DH^*) as a function of glucose inflow G_{in} and dilution rate D. The boundaries of the domains of coexistence and of existence of the sole producer are in dashed red and solid green lines, respectively. (B) For $G_{in} = 20$ g L^{-1}, productivity as a function of D for the consortium (filled circles), and for a producer growing in isolation (empty circles). The color code for coexistence (dashed red) or existence of the sole producer (solid green) is the same as in the main text. Vertical dashed lines indicate different productivity domains (see discussion in main text). (C) Same as B for the process yield $((DH^*)/(DG_{in}))$.

*Supporting Information of "Enhanced production of heterologous proteins by a synthetic microbial community: Conditions and trade-offs" (M. Mauri, J.-L. Gouzé, H. de Jong, E. Cinquemani)
