# Supplementary material for: Enhanced production of heterologous proteins by a synthetic microbial community: Conditions and trade-offs
Source: PLoS Comput Biol. 2020 Apr 13;16(4):e1007795. doi: 10.1371/journal.pcbi.1007795 (PMC7179936; doi:10.1371/journal.pcbi.1007795)
Supplement: S1 Text — (PDF) [file pcbi.1007795.s009.pdf]

S1 Text – Relation between biomass degradation constant and maintenance coefficient*

In the main text, we use the relation $k_{deg} = C_m Y_g$ to estimate the value of the biomass degradation constant via the maintenance coefficient C_m and the biomass yield coefficient of glucose Y_g. The degradation constant k_{deg} [h^{-1}] expresses the non-growth-related maintenance rate per unit biomass, the maintenance coefficient C_m [g gDW^{-1} h^{-1}] represents the rate of substrate taken up per unit biomass at zero growth rate and the yield coefficient Y_g [gDW g^{-1}] characterizes the conversion of substrate (glucose) into biomass. Experimentally, the maintenance coefficient C_m is obtained by extrapolating the experimental data via the relation introduced by Pirt [1].

$$\frac{dG}{dt} = -\frac{\mu}{Y_g}\, B - C_m\, B \tag{S1.1}$$

that relates the measured glucose uptake rate and the observed specific growth rate

$$\mu = \frac{1}{B}\frac{dB}{dt} \tag{S1.2}$$

during exponential growth on glucose in batch. According to the relations introduced in the main text, in our model in batch conditions and in the presence of glucose as the sole carbon source, Eqs S1.1 and S1.2 simplify to

$$\frac{dG}{dt} = -\, r_g^{up}\, B, \tag{S1.3}$$

$$\mu = Y_g\, r_g^{up} - k_{deg}. \tag{S1.4}$$

After substituting r_g^{up} from Eq. S1.4 into Eq. S1.3, we obtain

$$\frac{dG}{dt} = -\frac{\mu}{Y_g}\, B - \frac{k_{deg}}{Y_g}\, B. \tag{S1.5}$$

Compared to Eq. S1.1 this leads to the relation

$$k_{deg} = C_m Y_g \tag{S1.6}$$

between the measured maintenance coefficient and the biomass degradation constant, used for computing the latter in the main text. Fig. S1.1 represents graphically Eqs S1.1 and S1.5. It is important to note that both C_m and Y might depend on the carbon source, while k_{deg} is independent of the substrate since it corresponds to the (negative) growth rate in the absence of nutrients. We denote the dependence of C_m and Y on glucose and acetate by the subscripts $_g$ and $_a$, respectively. Therefore, for a bacterial culture growing on acetate, the degradation rate

$$k_{deg} = C_{mg}\, Y_g = C_{ma}\, Y_a \tag{S1.7}$$

has the same value for a culture growing on glucose and acetate. In the case of acetate, one can produce a figure similar to Fig. S1.1. The slopes and the intercepts of the fitted line will be different for acetate, but the extrapolated lines must both go through the point $(-k_{deg}, 0)$.

*Supporting Information of "Enhanced production of heterologous proteins by a synthetic microbial community: Conditions and trade-offs" (M. Mauri, J.-L. Gouzé, H. de Jong, E. Cinquemani)

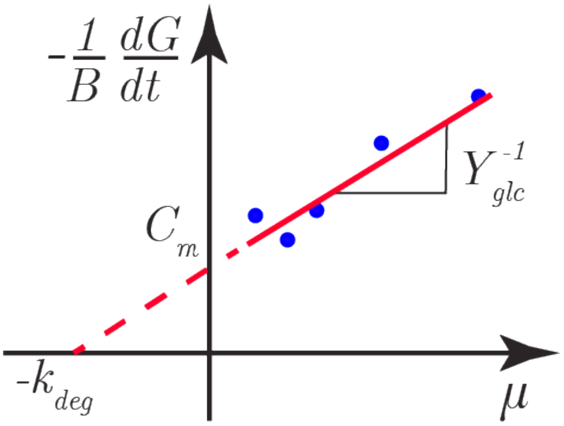

Figure S1.1: Relation between biomass degradation constant and maintenance coefficient. The maintenance coefficient C_m and the biomass degradation constant k_{deg} are obtained by extrapolating (dashed line) the data (dots) with Eqs S1.1 and S1.5, respectively, by measuring the specific substrate uptake rate $-dG/dt\,B^{-1}$ as a function of the specific growth rate $\mu = dB/dtB^{-1}$. The yield coefficient Y_g corresponds to the inverse of the slope of the interpolated relations (solid line).

Supporting references

[1] Righelato RC, Trinci APJ, Pirt SJ, Peat A. The Influence of Maintenance Energy and Growth Rate on the Metabolic Activity, Morphology and Conidiation of Penicillium chrysogenum. Microbiology. 1968;50(3):399–412.
