# Supplementary material for: Enhanced production of heterologous proteins by a synthetic microbial community: Conditions and trade-offs
Source: PLoS Comput Biol. 2020 Apr 13;16(4):e1007795. doi: 10.1371/journal.pcbi.1007795 (PMC7179936; doi:10.1371/journal.pcbi.1007795)
Supplement: S1 Software — The code is accompanied by a description, instructions of usage and a summary of the implemented model. (ZIP) [file pcbi.1007795.s010.zip › S1_Software/S1_Software_README.pdf]

S1 Software – MATLAB code used for model calibration, analysis and validation*

This document describes the MATLAB software implemented to calibrate, analyse and validate the models discussed in the main text. The software can be used in particular to reproduce the results in the main text. A summary of the model equations, model parameters and parameter values implemented by the code is reported in the last section of this document.

Quick usage

We assume the reader has unzipped the zipped folder containing this document, the file `main.m` and the subfolder `script`. Run MATLAB and set the current folder to the folder containing the script `main.m`. To generate all the figures of the main text, it suffices to call the function

```
main(run_estimation,run_heatmap,run_bootstrap)
```

from the MATLAB command window with appropriate parameter values. In particular, the call `main(1,1,1)` performs calibration, analysis, and validation of the model and shows the resulting figures. The (faster) call `main(2,2,2)` instead prepares and shows the figures from the previously stored results. The plotted figures report the reference to the corresponding figure of the manuscript* in their title. More options are discussed below.

Note: To avoid potential issues with the plotting, the reader is advised to close previously open figures and avoid interaction with the figures while the code is running.

Main

The function `main` accepts as input

- `run_estimation`: 0 skips parameter estimation from the data, a process that (depending on the computer) may take more than a minute, and uses previously computed estimation results; 1 performs the estimation, then saves and plots the results; 2 just loads and plots previously computed estimation results.
- `run_heatmap`: 0 skips the generation of the heat-maps $D - G_{in}$, a process that may take more than a minute; 1 computes the steady states, then saves and plots the results; 2 loads and plots previously computed results
- `run_bootstrap`: 0 skips the *a-posteriori* identifiability analysis of the estimated parameters, a process that takes from several minutes to more than an hour (depending on the machine); 1 performs the *a-posteriori* identifiability analysis via bootstrapping, then saves and plots the results; 2 loads a previously computed analysis and plots the results.

Initially, the `main.m` adds the paths of the subfolder `script` containing the scripts and the data. Then, it sequentially runs the functions

- `dynamics.m` to generate the time evolution of the variables in the bioreactor operating in continuous mode (demonstration figures not included in the manuscript)

*Supporting Information of "Enhanced production of heterologous proteins by a synthetic microbial community: Conditions and trade-offs" (M. Mauri, J.-L. Gouzé, H. de Jong, E. Cinquemani)

- `enjalbertData.m`, `basanData.m` and `bettenbrockData.m` to generate Figure 3A, B, C and D, respectively, regarding the model validation
- `DGinMap.m` to generate the steady-state analysis of the consortium in chemostat of Figures 5 and 6 of the main text (for several values of dilution rate D and glucose inflow G_{in}) for the case $Y_h = 0.2$ (right panels of Figure 5; the left panels of the same figure, corresponding to the case $Y_h = 0$, can be obtained by changing the value of Y_h in the function `parameters_values.m`)
- `fedbatch.m` to generate the Figure 7 of the main text regarding the protein production dynamics in fed-batch
- `bootstrap_fit.m` to generate Figure S1 in the supporting information of the *a-posteriori* identifiability analysis.

Each function can also be run separately by accessing the folder `script` and calling the corresponding `.m` file from the MATLAB command window, as described in the following.

Dynamics

The script `dynamics.m` in the folder `script` simulates the dynamics of the system under several conditions. `[T,X,dx,r] = dynamics(run_estimation,batchChemFed,producer,cleaner,hproteinP)` accepts as input

- `run_estimation`: 0 imports the already estimated parameters without plotting; 1 estimates the parameters by fitting the data and plots the results as in Figure 2 of the main text; 2 imports previously estimated parameter values and plots the corresponding estimation results
- `batchChemFed`: 0 simulates an experiment in batch; 1 in chemostat operating in continuous mode; 2 in fed-batch
- `producer`, `cleaner` and `hproteinP` can be set to 0 or 1 to simulate the presence (0) or absence (1) of the producer, cleaner and H protein production, respectively,

and gives as output

- `T`, the vector containing the time points of the simulation
- `X = [G,A,BP,H,BC]`, containing the time evolution of the variables glucose, acetate, producer biomass, H protein and cleaner biomass
- `dx = [dG,dA,dBP,dH,dBC]`, containing the time evolution of the state derivatives
- `r = [muP,muC,rgupP,raoverP,raupP,rH,rgupC,raoverC,raupC,kdeg]`, containing the time evolution of the rates: producer growth rate, cleaner growth rate, producer glucose uptake rate, producer acetate overflow rate, producer acetate uptake rate, heterologous protein production rate, cleaner glucose uptake rate, cleaner acetate overflow rate, cleaner acetate uptake rate and degradation rate.

The script `dynamics.m` calls

- `Model.m` that defines the system of differential equations to solve

- `parameters_values.m` containing the values of the parameters. If `run_estimation` is 1, it runs the parameter estimation by fitting the data in Figure 2 in the main text via `findParameters.m`. The latter minimizes squared fitting residuals by calling `minimizeChiSquare.m`. It also calls `pHratio.m`, which adapts the value of the parameter Θ_a to the hypothesized pH, as described in the main text. The data utilized are stored in the folder `script/data`. The values of the parameters are stored in the structure `parameters`
- `simulate_dynamics.m` and `plot_dynamics.m` , respectively solving the system of differential equations implemented in `Model.m` and plotting the results.

The structure `parameters` has fields

- `parameters.par` containing the parameter values
- `parameters.x0` containing the initial conditions of the state variables
- `parameters.plot` and `parameters.colors` defining several plotting options.

Model validation

The functions `enjalbertData.m`, `basanData.m` and `bettenbrockData.m` solve the system of differential equations defined in `Model.m`. Each of them loads the corresponding experimental initial conditions by running `parameters_values.m` and the publicly available experimental data (extracted from [1, 2, 3] from the `script/data` folder. Dynamics are simulated with `simulate_dynamics.m` or `generateDGinMap.m` (see below), and results are plotted as presented in Figure 3 of the main text.

Steady-state analysis

The function `DGinMap(run_heatmap,plot_heatmap,plot_rates)` generates the steady-state analysis of the consortium in chemostat operating in continuous mode as presented in Figures 5 and 6 of the main text, by varying the values of the dilution rate D and of the glucose inflow concentration G_{in}. If `run_heatmap` is set to 0, the generation of the heat-maps $D - G_{in}$ is skipped; if set to 1, steady states are computed numerically and results are plotted; if set to 2, results from a previous analysis are loaded and plotted. If `plot_heatmap` and `plot_rates` are set to 1, the function plots Figures 5A,B,C-F, and Figure 6A-C. The function `DGinMap`

- defines the differential equation of the model via `Model.m`
- loads the values of the parameters running `parameters_values.m`. First, it solves the system in the presence of the cleaner, then in the absence of the cleaner
- generates a grid of values of D and G_{in} with `create_heatmapparam`
- finds the steady state solution of the system of differential equations by numerical integration with the function `generateDGinMap`, via `simulate_dynamics.m`, using the MATLAB solver `ode15s`
- saves the solutions in the data files `solSSPC.mat` and `solSSPnoC.mat` in the case of cleaner presence and absence, respectively. The saved data contain the value of the dilution rate, glucose inflow concentration, variables at steady state and all the rates:
`solSS = [D,Gin,G,A,BP,H,BC,dG,dA,dBP,dH,dBC,muP,muC,rgupP,raoverP,raupP,rH, rgupC,raoverC,raupC,kdeg]`

- calls plot_DGinMap and DGinPlots to plot the map of stable solution as in Figure 5A,B, 6A and Figure 5C-F, Figure 6B,C, respectively.

Simulation in fed-batch

The script fedbatch.m generates Figure 7 of the main text regarding the protein production dynamics in fed-batch. It

- defines the differential equation of the model via Model.m
- laods the values of the parameters running parameters_values.m
- simulates the dynamics with simulate_dynamics in the presence and absence of cleaner strain
- plots the results with plot_dynamicsFedBatch.

Identifiability analysis

This analysis is performed by the function bootstrap_fit(run_bootstrap). If run_bootstrap is set to 0, the *a-posteriori* identifiability analysis is skipped; if set to 1, the analysis is performed via bootstrapping (see manuscript and code for details) and results are plotted as in supporting figure S1 Fig; if set to 2, a previously saved analysis is imported and the corresponding results are plotted. The script

- defines the differential equation of the model via Model.m
- loads the experimental data from the script/data folder
- generates 1000 new datasets
- fits the data with minimizeChiSquareBoot.m
- plots the result as in figure S1 Fig.

Requirements

The code was tested on typical installations of MATLAB 2017 and 2019 on Linux and Windows machines.

Variable	Unit	Meaning
G	g L^{-1}	Glucose concentration
A	g L^{-1}	Acetate concentration
B_P	gDW L^{-1}	Autocatalytic biomass concentration of the producer
H	gDW L^{-1}	Heterologous protein concentration
B_{totP}	gDW L^{-1}	Total biomass concentration of the producer
B_C	gDW L^{-1}	Biomass concentration of the cleaner

Table S1.1: List of variables. Meaning and units of the variables of the model of Eqs S1.1–S1.5 describing the producer strain, with rates as in Eqs S1.6–S1.8, and the cleaner strain, with rates as in Eqs S1.9–S1.11.

Summary of the model equations

The system of ordinary differential equations describing the model of the producer-cleaner consortium, as developed in the main text and implemented in the code, is given by

$$\frac{dG}{dt} = -r^g_{upP}\, B_P - r^g_{upC}\, B_C + \ D\,(G_{in} - G), \tag{S1.1}$$

$$\frac{dA}{dt} = \left(r^a_{overP} - r^a_{upP}\right)\, B_P + \left(r^a_{overC} - r^a_{upC}\right)\, B_C - D\,A, \tag{S1.2}$$

$$\frac{dB_P}{dt} = (1 - Y_h)\,\Big(Y_g\, r^g_{upP} + Y_a\,(r^a_{upP} - r^a_{overP})\Big)\, B_P - k_{deg}\, B_P - D\, B_P, \tag{S1.3}$$

$$\frac{dH}{dt} = Y_h\,\Big(Y_g\, r^g_{upP} + Y_a\,(r^a_{upP} - r^a_{overP})\Big)\, B_P - k_{deg}\, H - D\, H, \tag{S1.4}$$

$$\frac{dB_C}{dt} = \Big(Y_g\, r^g_{upC} + Y_a\,(r^a_{upC} - r^a_{overC})\Big)\, B_C - k_{deg}\, B_C - D\, B_C. \tag{S1.5}$$

The glucose uptake, acetate uptake, acetate secretion rates of the producer are expressed by

$$r^g_{upP}(G, A) = k_g\, \frac{G}{G + K_g}\, \frac{\Theta_a^n}{A^n + \Theta_a^n}, \tag{S1.6}$$

$$r^a_{upP}(G, A) = k_a\, \frac{A}{A + K_a}\, \frac{\Theta_g^m}{r^g_{upP}(G, A)^m + \Theta_g^m}, \tag{S1.7}$$

$$r^a_{overP}(G, A) = k_{over}\ \max(0, r^g_{upP}(G, A) - l). \tag{S1.8}$$

The glucose uptake, acetate uptake, acetate secretion rates of the cleaner are

$$r^g_{upC}(G, A) = k_{\Delta PTS}\, \frac{G}{G + K_g}\, \frac{\Theta_a^n}{A^n + \Theta_a^n}, \tag{S1.9}$$

$$r^a_{upC}(G, A) = k_a\, \frac{A}{A + K_a}\, \frac{\Theta_g^m}{r^g_{upC}(G, A)^m + \Theta_g^m} + k_{Acs}\, \frac{A}{A + K_{Acs}}, \tag{S1.10}$$

$$r^a_{overC}(G, A) = k_{over}\ \max(0, r^g_{upC}(G, A) - l). \tag{S1.11}$$

Table S1.1 and Table S1.2 list the meaning of the variables and of the parameters used in the equations. In view of these expressions and parameters, the growth rates of the producer and cleaner are given by

$$\mu_P = \Big(Y_g\, r^g_{upP} + \ Y_a\,(r^a_{upP} - r^a_{overP})\Big)\, \frac{B_P}{B_{totP}} - k_{deg},$$

$$\mu_C = Y_g\, r^g_{upC} + \ Y_a\,(r^a_{upC} - r^a_{overC}) - k_{deg},$$

Parameter	Unit	Meaning
D	h^{-1}	Dilution rate
G_{in}	g h^{-1}	Glucose inflow rate rate
k_g	1.53 g gDW^{-1} h^{-1}	Glucose maximal uptake rate
K_g	0.09 g L^{-1}	Glucose half-maximal saturation
Θ_a	0.52 g L^{-1}	Acetate inhibition constant
n	1	Acetate inhibition strength
k_{over}	0.17	Acetate overflow maximal overflow rate
l	0.7 g gDW^{-1} h^{-1}	Glucose uptake rate threshold for acetate overflow
k_a	0.97 g gDW^{-1} h^{-1}	Acetate maximal uptake rate
K_a	0.5 g L^{-1}	Acetate half-maximal saturation
Θ_g	0.25 g gDW^{-1} h^{-1}	Carbon catabolite repression inhibition constant
m	1	Carbon catabolite repression strength
Y_g	0.44 gDW g^{-1}	Biomass yield coefficient on glucose
Y_a	0.298 gDW g^{-1}	Biomass yield coefficient on acetate
Y_h	0.2	Heterologous protein yield constant
k_{deg}	0.0044 h^{-1}	Degradation rate
$k_{\Delta PTS}$	0.38 g gDW^{-1} h^{-1}	Glucose maximal uptake rate for ΔPTS strain
k_{Acs}	1.46 g gDW^{-1} h^{-1}	ACS acetate maximal uptake rate
K_{Acs}	0.012 g L^{-1}	ACS acetate half-maximal saturation

Table S1.2: List of parameters and their values. First block: Bioreactor parameters (any nonnegative values). Second block: Parameters of Eqs S1.1–S1.5 (producer and cleaner strains) and Eqs S1.6–S1.8 (producer strain). Third block: Parameters of Eqs S1.9–S1.11 (cleaner strain).

with $B_{totP} = B_P + H$.

Supporting references

[1] Enjalbert B, Millard P, Dinclaux M, Portais JC, Létisse F. Acetate fluxes in *Escherichia coli* are determined by the thermodynamic control of the Pta-AckA pathway. Sci Rep. 2017;7:42135.

[2] Steinsiek S, Bettenbrock K. Glucose transport in *Escherichia coli* mutant strains with defects in sugar transport systems. J Bacteriol. 2012;194(21):5897–908.

[3] Basan M, Hui S, Okano H, Zhang Z, Shen Y, Williamson JR, et al. Overflow metabolism in *Escherichia coli* results from efficient proteome allocation. Nature. 2015;528(7580):99–104.
